# Determinants of trust in healthcare facilities among community-based health insurance members in the Manna district of Ethiopia

**DOI:** 10.1186/s12889-023-15124-w

**Published:** 2023-01-25

**Authors:** Wakuma Akafu, Teferi Daba, Edosa Tesfaye, Firanbon Teshome, Tesfaye Akafu

**Affiliations:** 1 Department of Health Policy and Management, Faculty of Public Health, Institute of Health, Jimma University, Jimma, Oromia Ethiopia; 2 School of Public Health, Institute of Health, Wollega University, Nekemte, Oromia Ethiopia; 3 Department of Health, Behavior and Society, Faculty of Public Health, Institute of Health, Jimma University, Jimma, Oromia Ethiopia; 4 Department of Natural Resource Management, Institute of Agriculture and Veterinary Medicine, Jimma University, Jimma, Oromia Ethiopia

**Keywords:** Determinants, Trust, Healthcare, CBHI, Ethiopia

## Abstract

**Background:**

Low-income countries, including Ethiopia, face substantial challenges in financing healthcare services to achieve universal health coverage. Consequently, millions of people suffer and die from health-related conditions. These can be efficiently managed in areas where community-based health insurance (CBHI) is properly implemented and communities have strong trust in healthcare facilities. However, the determinants of community trust in healthcare facilities have been under-researched in Ethiopia.

**Objective:**

To assess the determinants of trust in healthcare facilities among community-based health insurance members in the Manna District of Ethiopia.

**Methods:**

A community-based cross-sectional study was conducted from March 01 to 30, 2020 among 634 household heads. A multistage sampling technique was used to recruit the study participants. A structured interviewer-administered questionnaire was used to collect the data. Descriptive statistics were computed as necessary. Multivariable linear regression analyses were performed, and variables with a p-value < 0.05 were considered to have a significant association with households’ trust in healthcare facilities.

**Results:**

In total, 617 households were included in the study, with a response rate of 97.0%. Household age (ß=0.01, 95% CI:0.001, 0.0013), satisfaction with past health services (ß=0.13, 95% CI:0.05, 0.22), perceived quality of services (ß= -0.47, 95% CI: -0.64, -0.29), perceived provider’s attitude towards CBHI members (ß = -0.68, 95% CI: -0.88, -0.49), and waiting time (ß= -0.002, 95% CI:- 0.003, -0.001) were determinants of trust in healthcare facilities.

**Conclusion:**

This study showed that respondents’ satisfaction with past experiences, older household age, long waiting time, perceived poor quality of services, and perceived unfavorable attitudes of providers towards CBHI members were found to be determinants of trust in healthcare facilities. Thus, there is a need to improve the quality of health services, care providers’ attitudes, and clients’ satisfaction by reducing waiting time in order to increase clients’ trust in healthcare facilities.

## Introduction

Trust is defined as the hopeful acceptance of a vulnerable condition under which the trustor believes that the trustee will protect the trustor’s best interests. This description highlights four important characteristics of trust: These characteristics include trust is a voluntary response to a set of expectations about how a trusted individual or institution will act towards others in the future; trust involves some degree of vulnerability and risk; and trust contains a relational concept rooted in the expectation that the other will have concern towards the best interests of others [1, 2]. There are different forms of trust. These include general and specific trust, rational-based and emotional-based trust, organizational trust, institutional trust, system trust, and personal trust. In addition to trust within and between individuals, there is also trust within and between organizations. Institutional trust is related to how it affects an institution’s guiding principles, routines, and controlling mechanisms. It is trust in norms and procedures, and not direct trust in people. Rather, it is a version of impersonal trust. Trust in the healthcare system is difficult to study because there is no consensus among scholars on how it should be conceptualized [3].

As part of its strategy to achieve universal health coverage (UHC), the government of Ethiopia has been implementing CBHI since 2011, in addition to other initiatives in the country. The CBHI was designed to cover citizens in the rural and informal sectors, estimated to be 85% of the Ethiopian population. It aims to cover 80% of districts enrolling at least 80% of eligible households [4, 5]. However, in most countries, including Ethiopia, enrollment in the schemes has been very low, and existing members have been dropping out of the scheme. Thus, the impact of CBHI on financial protection and access to needed healthcare is moderate [6–8].

Enrollment in the CBHI scheme is voluntary, which is an important feature that distinguishes CBHI from other health insurance schemes. This supports the hypothesis that trust in healthcare facilities may matter when people decide to enroll in and/or continue being members of the scheme. Health insurance is based on the fundamental idea that there is some uncertainty about future health outcomes and that the risk may be transferred to other parties in advance. In exchange for an agreement by the insurer to reimburse insurance to cover future losses, prepayment will be made to transfer this risk to the insurer and medical facilities. Therefore, the trust that members have in healthcare can play a decisive role in the local demand for increasing the uptake and ensuring the sustainability of the scheme [2, 9].

Low-income countries face substantial challenges in financing health services in order to achieve UHC coverage. Health services are unavailable and unaffordable for most of the poor in these countries. As a result, millions of people suffer and die from health and health-related conditions for which efficient and effective but underutilized interventions exist, particularly in settings that lack effective health insurance policies [10–13].

The role of trust in the healthcare systems of low- and middle-income countries (LMICs) has received little attention, even though trust is relevant across contexts [14]. One study found that trust in many institutions in high-income countries (HIC) was significantly reduced. For instance, in America, trust in people running medical institutions fell from 61% to 1974 to 37% in 2018, and trust in the healthcare system dropped from 80% to 1975 to 38% in 2019 [15]. Building trust is important for maintaining the CBHI scheme and improving services for health insurance members. The patient-doctor relationship relies on patients’ confidence that physicians have their best interest in the heart, especially when the patient is particularly vulnerable. Similarly, trust between CBHI members and healthcare organizations with which they work and collaborate is essential for the efficient functioning of the healthcare system and for reforms like CBHI [3].

Public confidence in medical facilities has been said to have declined for various reasons [16]. A common misconception is that distrust in healthcare is primarily related to past atrocities on human beings, such as Tuskegee, Sims, and others, but the main condemnation of these entities ignores everyday alleged acts. Studies have identified factors thought to contribute to the declining trust in healthcare facilities, including negative past experiences, providers’ attitudes, and lack of a system in place to make them return to the same health facility after an experience in which they lost their trust [17, 18], highly shown conflicts of interest between clinicians and device manufacturers, a rise in managed care and related financial incentives, and a very short period of time for communication and fragmentation of the patient-clinician connection [19–21].

Yet, there is an opportunity for healthcare institutions to actively rebuild and get back the trust of their communities. Rebuilding trust is essential because the link between trust and health outcomes is well recognized [18]. Trust appears to be necessary when there is uncertainty and a level of risk, be it low, medium, or high. This element of risk appears to be derived from an individual’s uncertainty regarding the motives, intentions, and future actions of another on whom the individual is dependent. [16].

Recent studies have highlighted the potential value of trust in understanding the performance of healthcare organizations and public health institutions, as CBHI members’ trust in health facilities has been reported to facilitate insurance enrollment and sustainability decisions [5, 9, 22, 23]. The absence of trust and trust crisis in healthcare systems among insurance members is associated with rapidly rising costs, a large number of uninsured people, less doctor-patient interactions, poor clinical relationships that exhibit less continuity with existing reforms such as CBHI, reduced adherence to recommendations and guiding principles of the CBHI scheme, worse self-reported health, reduced utilization of health care services, decreased CBHI enrollment, and increased dropout from the scheme, which in turn affect the sustainability of the CBHI scheme [24, 25].

Various studies have examined individual-level trust, between patients and providers [14, 26–30]. Studies have been conducted on the factors affecting trust among health insurance members in the CBHI scheme [2, 9, 24]. However, very little research has focused on healthcare experiences that are likely to degrade trust in healthcare institutions [16, 31]. Still, health facilities are too far from understanding, protecting, and returning trust in healthcare systems, not only for the effective functioning of healthcare systems but also for the public best interest in general. Identifying and managing mistrust in healthcare systems could contribute to improving the effectiveness and efficiency of health services while protecting community health [32].

Studies have emphasized that much work needs to be done regarding the determinants of trust in hospitals and other medical institutions to end up with a clear imbalance between the importance of trust in the functioning of healthcare systems and the priority given to research on trust. Thus, a more vibrant exchange of knowledge among researchers, policymakers, and managers is needed [17, 32].

To the best of our knowledge, little is known about the determinants of trust in healthcare facilities among CBHI members in Ethiopia. Therefore, this study was designed to assess the determinants of trust in healthcare facilities among CBHI members in the Manna district of Ethiopia, which adds input for program designers, healthcare managers, health insurance agencies, and health workers to generate and earn back trust within and throughout the health system.

## Methods and materials

### Study design, setting and period

A community-based cross-sectional study was conducted from March 1 to March 30, 2020, in the Manna district, among CBHI members during the year 2016–2020. Manna district is located 382 km southwest of Addis Ababa, (the capital city of Ethiopia). The Manna district is one of the four districts where CBHI was first introduced in the Jimma zone. CBHI registration began in this district in 2014. Service utilization began in 2016. The number of households enrolled in the district scheme is 10,713. According to a report obtained from the Manna District Health Office, the total population of the district was 205,497, of which 42,812 were households. Among the total population of the district, the majority (82.6%) and 89% were Oromo by ethnic group and native Afan Oromo speakers, respectively. There are 26 kebeles and four health centers in the district [33].

### Population and sampling techniques

All households that had enrolled in the health insurance scheme in the district were the source population. The study populations were selected households that were members of the CBHI and fulfilled the inclusion criteria. Households ever registered in the CBHI scheme of the Manna district were included in the study, whereas households with less than one year of membership were excluded from the study.

The sample size was determined using a single population proportion formula considering the following assumptions: 95% confidence level (1.96), 50% proportion of trust in the health facility was considered because no study found that addressed the objective of this study, 5% margin of error, 1.5 design effect, and 10% nonresponse rate. Accordingly, the final sample size becomes 634.

The Manna district was chosen purposively because it is one of the districts in the Jimma zone, where health insurance was first tested as a pilot and scale-up was made. A multistage sampling technique was employed to select the study participants. First, 26 villages in the district were stratified into urban [1] and rural [25] villages. The urban village was purposively selected for representativeness. Eight of the 25 rural villages were selected using a simple random sampling technique. The calculated sample size was proportionally allocated to each of the selected villages. Study participants were selected using a computer-generated simple random sampling technique. A list of household heads enrolled in the scheme was obtained from the district health office. Their usual place of residence was identified in collaboration with village leaders.

**Fig. 1:**
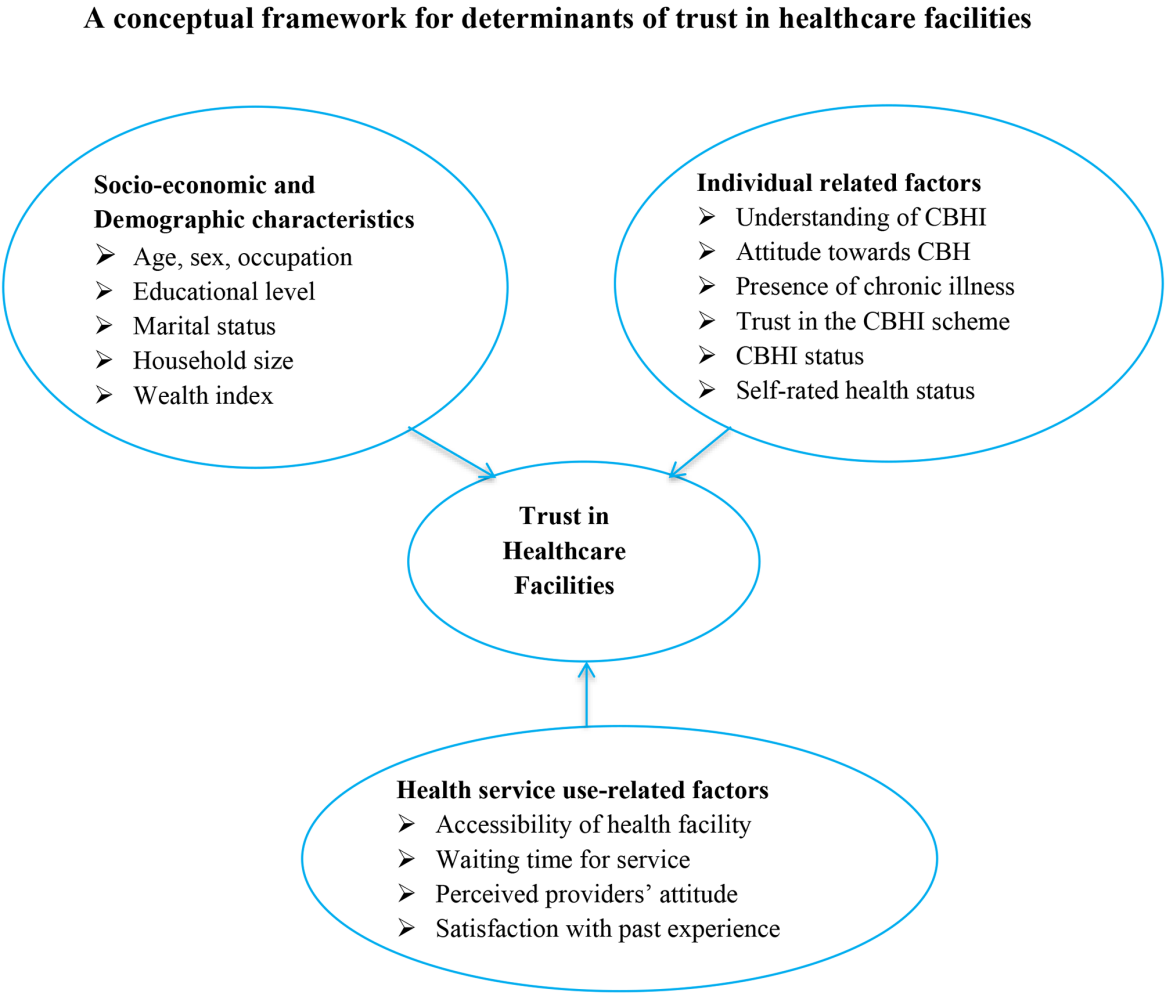
Conceptual framework for determinants of trust in healthcare facilities among community-based health insurance members

### Data collection tool and procedures

An interviewer-administered structured questionnaire was adapted from relevant literatures [2, 9, 23, 34]. The questionnaire had five major sections: socioeconomic and demographic characteristics, CBHI status of household heads, individual or household-related variables, CBHI-related factors, and health service-related factors. The questionnaire was translated from English into the local language Afan Oromo by a language expert from Jimma University. It was then back-translated into English by another independent person to ensure consistency. Finally, the tool was pre-tested in a nearby district, Seka Chekorsa District, two weeks ahead of the actual data collection period, and necessary modifications, such as unclear or vague questions and incorrect skip patterns, were made accordingly. Both data collectors and supervisors were involved in the pre-test activity. Cronbach’s alpha was used to assess internal consistency of the variables. Trust in health care institutions was the outcome variable, whereas socioeconomic and sociodemographic factors (household head’s age, occupation, sex, marital status, educational status, household size, and wealth index), individual-related factors (CBHI status, attitude towards CBHI, understanding of CBHI, recent illness episode, chronic disease, self-rated health status, perceived quality of service, and trust in the CBHI scheme), and health service use-related factors (waiting time for service, availability of service, accessibility of service, perceived providers’ attitude, and satisfaction with past experience) were independent variables for the study (Fig. 1).

Six data collectors (diploma nurses) and two supervisors (Bachelor of Science in public health officers) were recruited for data collection based on their previous experiences and fluency in the local language. One-day intensive training was given to the data collectors and supervisors on the objective of the study, data collection tools, approach to the interviewees, and confidentiality and privacy of the respondents.

### Operational definitions and measurements

#### Dropout to CBHI

Households that had ever enrolled in the CBHI scheme but discontinued their membership or failed to renew the contract of insurance after enrolling at least for one year in an allotted grace period (December to February) for renewal [35].

#### Renew

Households who had had CBHI for more than one year and who were enrolled at the time of data collection were classified as renewed, and households enrolled in the first year of operation, dropped in the second year, and enrolled again in the third year of operation were considered as ‘renewal’.

#### Trust in the health care facilities

This is measured by ten items with a 5-point Likert scale ranging from 1 (strongly disagree) to 5 (strongly agree). Factor scores were computed using principal component analysis (PCA). The overall Kaiser-Meyer-Olkin (KMO) value was 0.5, indicating that the sample was adequate for factor analysis. Indeed, Bartlett’s test of sphericity was significant, showing that the correlation between the items was sufficiently large for factor analysis [36]. Finally, one component was extracted from one eigenvalue, with a total variance of 69.5%. This variable was treated as a continuous variable in the analysis. The internal consistency of the variables was checked using Cronbach’s alpha, which was found to be 86.9%.

#### Perceived quality of health services

This variable was measured on a 5-point Likert scale ranging from 1 (very poor) to 5 (very good). Subsequently, three dummy variables (poor, medium, and good) were created for analysis.

#### Attitude towards CBHI scheme

This was measured by 10 items on 5 points Likert scale ranging from 1 (strongly disagree) to 5 (strongly agree). The assumption of summated scales was employed to examine the overall score that represents the respondent’s position on the continuum of favorableness toward the CBHI. Thus, based on this continuum of favorableness, it was categorized into three dummy variables unfavorable 10–29, neutral 30, and favorable attitude 31–50 [37].

#### Understanding of CBHI

Households were asked 10 items about CBHI, then respondents were classified as having a ‘high level of understanding’ if the household answered greater than or equal to mean and otherwise ‘low level of understanding‘ [34].

#### Household wealth index

Household assets were collected based on the types of consumer goods they owned, such as domestic animals, durable/non-productive assets, productive assets, housing utilities, and other household materials. Factor scores were then derived using PCA, and composite scores were categorized into five quantiles. The first 20% quantile was classified as the poorest, whereas the last 20% quantile was considered the richest. The KMO measure of sample adequacy was 0.747 with a significant Bartlett’s test of sphericity. The total variance explained by these variables was 66.7% [38].

#### Trust in CBHI scheme

Participants were asked five items on a 5-point Likert scale ranging from 1 (strongly disagree) to 5 (strongly agree). The factor scores were computed using PCA. This was treated as a continuous variable in the analysis [9].

#### Perceived Provider’s attitude

It is measured by 10 items on 5 points Likert scale ranging from 1 (strongly disagree) to 5 (strongly agree). The factor score was computed using PCA, and the extracted factor score was ranked based on percentile and then categorized into three dummy variables for analysis of whether a member perceives that the providers have an unfavorable, neutral, or favorable attitude towards the CBHI member.

### Data processing and analyses

After checking for completeness and consistency, the collected and coded data were cleaned, checked, and entered into Epi Data Manager Version 3.1 software, and then exported to SPSS version 26 for analyses. The frequency distributions of all variables were examined to check for data entry errors. For PCA, extractions with eigenvalues greater than one and varimax rotation, methods were employed. The KMO measure of sample adequacy above 0.5 with significant Bartlett’s test of sphericity was used. Finally, the component extracted with a total variance of ≥ 60% was used for the analyses. The extracted factors were renamed according to the items contained in them. Items with insignificant loadings (loading below 0.40) and cross loadings were excluded from the analyses. An eigenvalue greater than one decision rule was used to determine the appropriate number of factors to be extracted. Items with Cronbach’s alpha values greater than 0.7 extracted from each of the scales were used in subsequent analyses. The factor score computed for the outcome variables was treated as a continuous variable for multiple linear regressions [36].

Descriptive analyses were used to describe trust in healthcare institutions and are presented in terms of frequencies, percentages, figures, and tables, as necessary. Simple linear regression analysis was performed to select candidate covariates for multivariable analysis. Then, covariates with a p-value < 0.1 in a simple linear regression analysis were considered as candidates for the multivariable analyses model. A backward method of multivariable linear regression analysis was performed to control for possible confounding factors and identify possible determinants of trust in healthcare institutions. The 95% confidence intervals and beta coefficients were calculated and used to describe the statistically significant covariates. A p-value < 0.05 was considered a statistically significant determinant of trust in healthcare institutions. All necessary assumptions for multivariable linear regression were checked before the analysis was performed. Multicollinearity between independent variables was checked using the variance inflation factor (VIF), and the maximum VIF was found to be 2.23 [39, 40].

### Ethical consideration

Ethical clearance was obtained from the Jimma University Ethical Review Board (Ref. No: IRB00052/2020). This study was conducted in accordance with the principles of the Declaration of Helsinki. All study participants were well informed about the purpose of the study, and written informed consent was obtained from study participants who were able to read and write before the interviews. As the questionnaire was interviewer-administered, the interviewer read the consent form for participants who were unable to read and write, and a finger signature was obtained before the interviews. The study participants’ confidentiality was secured, and no personal identifiers were used in the data collection tools, and codes were used instead. All paper- and computer-based data were stored in protected and safe locations. The recorded data were not accessed by a third person except for the research team, and data sharing was enacted based on the ethical and legal rules of data sharing.

## Results

### Background characteristics of the study respondents

The total number of households that participated in the study was 617, with a response rate of 97.3%. The mean age of the households was 44.71(± 11.17) years. The majority (88.2%, 84.6%, and 85.6%) were rural by residence, male by sex, and Oromo by ethnicity, respectively. Nearly half, (50.7%) of respondents had no formal education. The mean family size of the households was 5.54(± 1.87) (Table 1).

**Table 1:** Socio-economic and sociodemographic characteristics of study respondents in Manna district, Jimma zone, southwest, Ethiopia, 2020

Variables	Frequency	Percent
**Age category**		
18–30	62	10
31–40	205	33.2
40–50	197	31.9
≥ 51	153	24.8
**Ethnic group**		
Oromo	528	85.6
Amhara	13	2.1
Dawuro	44	7.1
Other	32	5.2
**Sex**		
Male	522	84.6
Female	95	15.4
**Residence**		
Rural	544)	88.2
Urban	73	11.8
**Marital status**		
Married	553	89.6
Divorced	28	4.5
Separated	18	2.9
Widowed	18	2.9
**Religion**		
Muslim	509	82.5
Orthodox	35	5.7
protestant	73	11.8
**Distance from health facilities**		
< 30 min	185	30
≥ 30 min	432	70
**Educational level**		
Unable to read and write	199	32.3
Able to read and write	114	18.5
Primary education	181	29.3
Secondary and above	123	19.9
**Household size**		
≤ 5	301	48.8
> 5	316	51.2
**Respondents’ main Occupation**		
Farmer	451	73.1
Merchant	52	8.4
Daily laborer	74	12
Petty traders and other	40	6.5
**Households’ Wealth index**		
Poorest	121	19.6
Poor	124	20.1
Middle	125	20.3
Rich	123	19.9
Richest	124	20.1

### Respondents’ CBHI status and perceived quality of health services

**Fig. 2:**
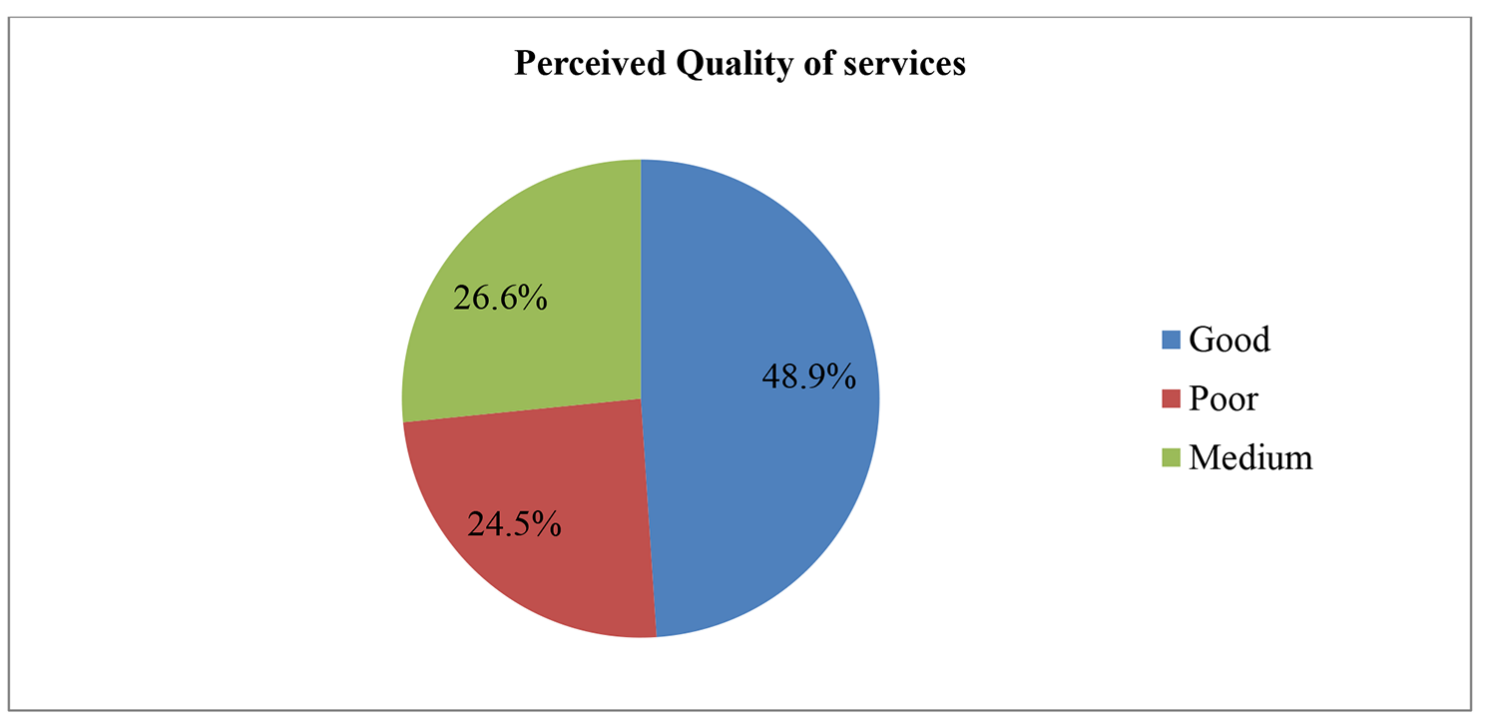
Perceived Quality of health services among ever enrolled in the CBHI scheme of Manna District, Jimma Zone, 2020

Concerning the CBHI status of the study participants, 420(68.1%) members who were enrolled in the CBHI scheme of Manna district renewed their CBHI membership at the time of the study. In relation to the perception of quality of services, perceived quality of health services has a strong influence on trust in healthcare facilities. As identified by this study, there is a significant difference in trust in healthcare facilities among those who perceived good quality of services and poor quality of services. Accordingly, as shown in Fig. 2, nearly half (48.9%) of the respondents perceived that the quality of services they were taking from health facilities was good, whereas approximately one-fourth (26.6%) of them perceived that they were taking poor quality health services from health facilities (Fig. 2). Regarding waiting time to get services, around 476 (77.1%) respondents waited for more than or equal to thirty minutes to get services.

### Understanding level and attitude of CBHI members toward the CBHI Scheme

**Fig. 3:**
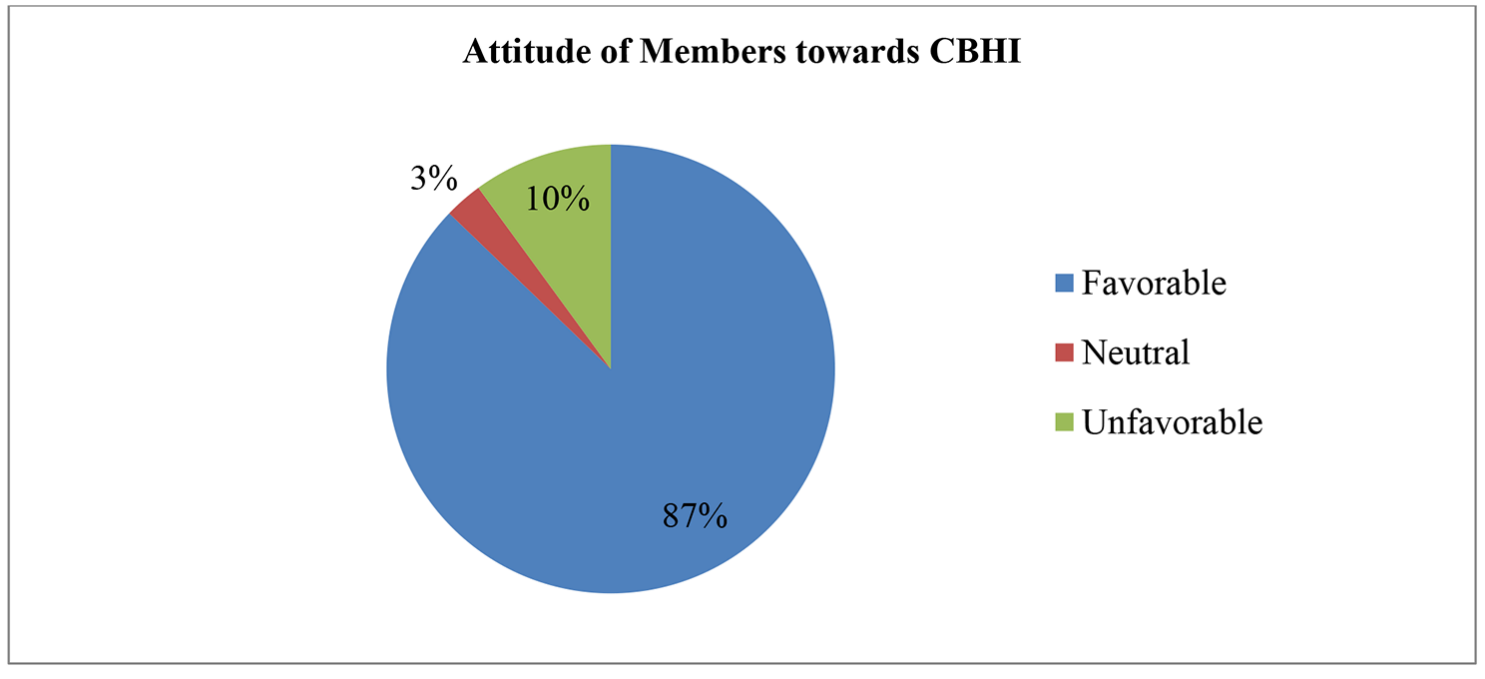
Attitude of CBHI members towards CBHI Scheme among those ever enrolled in the CBHI scheme of Manna District, Jimma Zone, 2020

In relation to the attitude of CBHI members towards the CBHI scheme, irrespective of their CBHI status, 538 (87%) participants had a favorable attitude towards the CBHI scheme, whereas 62 (10%) of the study respondents had an unfavorable attitude towards the CBHI scheme (Fig. 3). Regarding the understanding level of CBHI members, 439(71.2%) respondents had a high level of understanding of the guiding principle and implementation procedure of the CBHI scheme. Perceived providers’ attitudes toward CBHI members also matter for CBHI members to trust health facilities. According to this study, only 191(31.1%) respondents perceived that health providers had favorable attitudes toward CBHI members. However, 208(33.7%) respondents perceived that health providers had an unfavorable attitude toward CBHI members.

### Determinants of trust in the healthcare facilities

A total of nine variables (household heads’ age, trust in the CBHI scheme, satisfaction of members with past experience, perceived service quality, perceived provider’s attitude toward CBHI members, educational level of the respondents, distance to the nearest health facilities by foot, waiting time to receive services, and household wealth index) had a p-value < 0.1 and taken as candidates for the multivariable linear regression model.

After controlling for confounding factors in multivariable regression, five variables (respondents’ age, perceived quality of health services, satisfaction with health services, perceived providers’ attitude toward CBHI members, and waiting time to get services) remained statistically significant with respondents’ trust in health care facilities. This study showed that household age has a positive relationship with trust in healthcare facilities. For a one-year increase in the respondents’ age, the scores of trust in health facilities increased by 0.01 [adjusted ß = 0.01 (95% CI: 0.001, 0.0013; p = 0.023)] holding other variables constant. This finding showed that CBHI members’ satisfaction with what they had experienced at healthcare institutions was positively associated with trust in healthcare facilities. Accordingly, for one unit increase in the satisfaction score, the trust score was increased by 0.13 [adjusted ß = 0.13 (95% CI: 0.05, 0.22; p = 0.002)], keeping other independent variables constants. The perceived quality of services also determines trust in healthcare facilities. The trust score among households that perceived poor quality of services decreased by 53% [adjusted ß = -0.47 (95% CI: -0.64, -0.29; p < 0.001)] compared to those who perceived good quality of services.

The way the CBHI members perceived, the attitude that health professional had towards CBHI members also determine the trust that members had in the healthcare facilities. As shown in Table 2, healthcare providers’ perceived unfavorable and neutral attitudes towards CBHI members had a negative relationship with trust in healthcare institutions. Accordingly, the trust score decreased by 32% [adjusted ß = -0.68 (95% CI: -0.88, -0.49; p < 0.001)] and 75% [adjusted ß= -0.25 (95% CI: -0.42, -0.09; p < 0.001)] among households who perceived that health professionals had an unfavorable and neutral attitude towards CBHI members, respectively, compared to their counterparts. Another finding of this study was that waiting time was significantly negatively associated with trust in healthcare institutions. This finding also showed that for a minute increase in waiting time to obtain health services, trust in the healthcare institution decreased by -0.002 [adjusted ß = -0.002, 95% CI: -0.003, -0.001; p < 0.001)], keeping other variables constant (Table 2).

**Table 2:** Determinants of households’ trust in health care facilities among CBHI members in Manna district, Ethiopia, 2020

Variables	Simple linear regression		Multivariable linear regression	
	B	95% CI B	***B***	**95%CI** ***B***
**Household heads’ age**	0.01	0.003 to 0.017	0.007	**0.001 to 0.0013** ^*^
**Trust in the CBHI scheme**	0.36	0.29 to 0.44	0.062	-0.024 to 0.15
**Satisfaction of members with past experience**	0.44	0.37 to 0.51	0.13	**0.05 to 0.22** ^*^
**Perceived service quality**				
Good	**Ref**			
Poor	-0.97	-1.13 to -0.80	-0.47	**-0.64 to -0.29** ^**^
Medium	0.07	-0.12 to 0.25	-0.11	-0.28 to 0.06
**The educational level of the respondents**				
Unable to read and write	**Ref**			
Able to read and write	0.13	-0.08 to 0.33	0.16	-0.007 to 0.33
Primary	0.05	-0.13 to 0.22	0.004	-0.15 to 0.16
Secondary and above	0.19	-0.003 to 0.39	0.004	-0.19 to 0.20
**Perceived provider’s attitude toward CBHI members**				
Favorable	**Ref**			
Unfavorable	-0.97	-1.12 to -0.824	-0.68	**-0.88 to -0.49** ^**^
Neutral	0.23	0.07 to 0.39	-0.25	-0.42 to -0.09^*^
Distance to the nearest HF on foot (in minutes)	-0.004	-0.006 to -0.002	-0.001	0.001 to 0.91
Waiting time to get services (in minutes)	-0.005	-0.006 to -0.004	-0.002	**-0.003 to -0.001** ^**^
**Household’s wealth index**				
Poorest	Ref			
Poor	0.038	-0.158 to 0.235	0.058	-0.104 to 0.220
Middle	0.026	-0.172 to 0.224	0.059	-0.119 to 0.237
Rich	0.041	-0.156 to 0.239	0.077	-0.090 to 0.244
Richest	0.015	-0.18 to 0.213	0.081	-0.127 to 0.290

**and *Denote statistical significance at the 1% and 5% levels, respectively.

Abbreviation: Ref = Reference group, HF: Health facility.

The amount of trust in healthcare institutions explained by the independent variable was shown by the adjusted R-square, and it was found to be 33.2%.

## Discussion

Trust that CBHI members have in healthcare facilities plays a paramount role in addition to the trust they have in the scheme. However, other than highlighting the importance of trust, determinants of trust in healthcare facilities among CBHI members have not been studied yet in Ethiopia for which this paper was primarily designed. This study found that respondents’ age, perceived quality of health services, satisfaction with health services, perceived providers’ attitude toward CBHI members, and waiting time to get services were significant determinants of trust in healthcare facilities.

This study showed that household age has a positive relationship with trust in healthcare facilities. This finding is in line with a study conducted on trust in physicians and medical institutions, which revealed that patient characteristics are not strong predictors of trust in medical institutions. Excluding age, studies have found inconsistent, weak, or no relationship between trust and most demographic characteristics. However, age had a modest positive correlation with trust in medical institutions, which supporting the findings of this study [1]. A possible justification for this finding could be that, as the age of the household increases, the susceptibility to different diseases also increases as their immunity decreases and is more likely to be prone to sickness than younger individuals. As they continuously and consistently visit health facilities, they could start to have confidence in healthcare institutions.

Another issue revealed by this study was that CBHI members’ satisfaction with what they had experienced at healthcare institutions was positively associated with trust in healthcare institutions. This finding is supported by a study conducted in Bangladesh and Ethiopia [9, 41]. These studies have found a strong correlation between trust and satisfaction. However, the question is whether satisfaction with the care received drives trust or whether trust in healthcare facilities drives satisfaction. Insight comes from one study concluded that trust is the primary driver based on changes in trust and satisfaction [42]. This finding suggests that satisfaction with healthcare services plays an important role in designing future health insurance programs and promoting client-oriented health services.

According to this study, another factor that determines trust in healthcare institutions is the perceived quality of the services that members receive from health facilities. Accordingly, the trust score decreased by 0.47 among households that perceived poor quality of services compared to households that perceived good quality of services. A study conducted in Burkina Faso, Senegal, and Ethiopia revealed a negative relationship between membership renewal and poor perceived quality of services [5, 8, 43]. A study conducted in Ethiopia showed that trust in the CBHI scheme increased by 0.15 among households who perceived good quality of services compared to households who perceived poor quality of services [9]. Based on the above findings, we can conclude that perceived quality of service affects the trust in healthcare facilities. This could be justified by expanding access to services for members or holding the line on costs is insufficient if one’s trust in the quality of healthcare services is low. Perceptions of the poor quality of health services may discourage members from using available services and healthcare financing reforms like CBHI. If the system cannot be trusted to guarantee a threshold level of quality, it will remain underutilized and used as a measure of the last way out, bypassed, or used only for minor ailments. This finding implies that, in addition to increasing access to health services, ensuring the quality of these services in the best interests of the beneficiaries helps the sustainability of the CBHI scheme in a resource-limited setting like Ethiopia.

This study revealed that the way the CBHI members perceived the attitude that the health professionals had towards CBHI members also determined their trust in healthcare facilities. As shown by this study, perceived unfavorable and neutral attitudes of providers towards CBHI members had a negative relationship with trust in healthcare institutions. This finding is in line with a study conducted on trust in healthcare institutions in South Africa [14]. This finding can be explained by the fact that trust in healthcare is commonly influenced by patient judgments about provider attitudes, as well as technical competence. The acts of being courteous or rude, treating some people preferentially over others, demanding bribes, giving the patient too little time, and listening to the patient’s explanation of their complaint influence patient judgments of whether the provider cares for their best interests. CBHI members who lost trust in their healthcare provider would not return to the same provider or healthcare facility where they had experienced a loss of trust, which in turn declines their trust in healthcare facilities.

Another finding of this study was that waiting time (length of time between registration and use of the service) was significantly negatively associated with trust in healthcare institutions. This finding was in line with the study conducted in Rwanda, which showed that there is a linear relationship between adherence to the CBHI scheme and dissatisfaction of outpatients with regard to long waiting times to be seen by a medical care provider [44]. A study conducted in Ghana identified long waiting times as a barrier to enrollment [35]. Another study showed that a long waiting time is also considered a contributing factor to low enrollment in the CBHI scheme in developing countries [45]. This can be further explained as a failure of healthcare facilities to respond to consumer demands and beneficiary’s affairs in a timely manner might affect the faith and confidence that beneficiary have in healthcare facilities, which in turn imposes a lack of trust in healthcare facilities.

### Strengths and limitations of the study

To the best of our knowledge, this is the first study of its kind in the study area, as well as at the national level, and is believed to provide useful information for health facilities and the existing CBHI scheme. Furthermore, the study was community-based and a representative study population was used, which enabled the generalizability of the study findings to the source population. As a limitation, due to the scarcity of research in the title area old references were used. In addition, sometimes an individual might respond to the questions in a way that is acceptable to many other than telling their true feelings, which might prone this study to social desirability bias. This study would have benefited had it been supported by qualitative data collection, as the method of data collection through a structured questionnaire might not allow respondents to give their perceptions and motivations regarding the subject of trust.

## Conclusions

This study assessed the rapidly developing body of work on trust in medical settings, determinants of trust in healthcare facilities among households enrolled in the CBHI scheme. Trust in healthcare facilities is important for the CBHI scheme to enroll and maintain its members, given the amount of risk inherent in the nature of insurance schemes. Therefore, this study revealed that CBHI members’ satisfaction with what they had experienced at healthcare institutions and household age were positively associated with trust in healthcare institutions. Whereas, long waiting times, perceived poor quality of services, and perceived unfavorable and neutral attitudes of healthcare providers towards CBHI members were determinants that had a negative relationship with trust in healthcare facilities and determinants that had been degrading trust in healthcare institutions among ever enrolled households to the CBHI scheme, for which great attention needs to be considered to regain and maintain trust in healthcare that results in a reduction of fear and uncertainty about the future.

Thus, we strongly recommend that the existing health system should prioritize rebuilding and protecting trust in healthcare facilities by reducing waiting time for services, increasing the satisfaction of members with services they are taking at health facilities, and working on the attitude that healthcare workers have for insurance members while maximizing the overall quality of services to the best interests of the beneficiaries. This, in turn, supports earning, building, and preserving trust in healthcare institutions because if we wait until trust in healthcare facilities is eroded or even broken before giving its analysis and sufficient priority it may be too late and too costly to rebuild it again.

## Data Availability

The datasets generated during the current study are available from the corresponding author upon reasonable request.
